# Effects of less invasive surfactant administration versus intubation-surfactant-extubation on bronchopulmonary dysplasia in preterm infants with respiratory distress syndrome: a single-center, retrospective study from China

**DOI:** 10.1186/s12890-022-02270-x

**Published:** 2022-12-05

**Authors:** Chun-cai Xu, Ying-ying Bao, Jing-xin Zhao, Ke Cheng, Ling Sun, Jing-yuan Wu, Ming-yuan Wu, Jia-jun Zhu

**Affiliations:** Department of Neonatology, Women’s Hospital School of Medicine, Zhejiang University, Hangzhou, China

**Keywords:** Bronchopulmonary dysplasia, Less invasive surfactant administration, Intubation-surfactant-extubation, Preterm infants

## Abstract

**Background:**

This study evaluated the effects of less invasive surfactant administration (LISA) and intubation-surfactant-extubation (InSurE) on bronchopulmonary dysplasia (BPD) in preterm infants with respiratory distress syndrome (RDS).

**Methods:**

Neonates with respiratory distress syndrome requiring surfactant, with gestational age < 32 weeks and birth weight < 1500 g admitted to our neonatal intensive care unit from January 2018 to December 2019, were retrospectively analyzed. LISA and InSurE were used independently. The incidence of BPD at 36 weeks postmenstrual age, pre-discharge mortality, and need for mechanical ventilation (MV) within 72 h of birth were compared between LISA and InSurE group. Secondary outcomes including necrotizing enterocolitis requiring surgery, retinopathy of prematurity ≥ stage 3, patent ductus arteriosus requiring medical therapy or surgery, and length of hospitalization were analyzed.

**Results:**

Among the 148 included neonates, there were 46 and 102 infants in LISA group and InSurE group, respectively. There were no significant differences in BPD incidence, the severity of BPD at 36 weeks postmenstrual age, and the rate of MV within the first 72 h after birth between the two groups (*P* > 0.05, respectively). The incidences of necrotizing enterocolitis requiring surgery, retinopathy of prematurity ≥ stage 3, patent ductus arteriosus requiring medical therapy or surgery, and length of hospitalization did not differ significantly between the two groups (*P* > 0.05, respectively).

**Conclusions:**

For surfactant administration among preterm infants with respiratory distress syndrome, LISA did not decrease bronchopulmonary dysplasia and severity of BPD at 36 weeks postmenstrual age. The benefits of LISA would require further evaluations.

## Background

Neonatal respiratory distress syndrome (RDS) is a common disease in premature infants, specifically in early preterm infants, due to surfactant deficiency. Surfactant replacement therapy has been widely used for infants with RDS, because it reduces mortality and the incidence of bronchopulmonary dysplasia (BPD) [1]. Intubation-surfactant-extubation (InSurE) is the most applied method of surfactant delivery. In recent years, a novel method of surfactant administration, called as less invasive surfactant administration (LISA), has become more popular and fashionable in an effort to reduce injury to lungs of early preterm infants [2, 3]. There are two main changes in LISA method, compared with InSurE. One is that infants are intubated with a thin catheter instead of a tracheal tube to deliver the surfactant; the other is that infants are supported by continuous positive airway pressure with spontaneous breaths instead of positive pressure ventilation during the surfactant administration process. It has been shown, that LISA may reduce combined incidence of mortality and BPD in preterm infants at 36 weeks gestational age (GA) in the recent systematic review [4]. However, differences in the gestational age of infants and indications for surfactant replacement in previous studies have weakened the evidence [5–9]. Furthermore, there is still no consensus regarding the efficacy of the LISA procedure, which may hamper its worldwide acceptance. We have collated our experience in this issue to confirm the efficacy and competitive strength of LISA.

This study analyzed LISA’s effects on the incidence of BPD and mortality in preterm infants with RDS, compared with the traditional InSurE method.

## Methods

This retrospective observational study was performed in the tertiary neonatal intensive care unit (NICU) of the Women’s Hospital, School of Medicine, Zhejiang University, between January 2018 and December 2019. The requirement for informed consent was waived due to the study's retrospective nature. The participants were classified into two groups based on the method of surfactant administration during the study period: (1) LISA group, in which infants were treated with surfactants by the LISA method, and (2) InSurE group, in which infants were treated with surfactants by the traditional method.

The inclusion criteria were as follows: (1) having RDS with the gestational age < 32 weeks and the birth weight < 1500 g; (2) having spontaneous breathing and being stable on nasal continuous positive airway pressure (nCPAP) with a pressure of 5–8 cmH_2_O; (3) requiring fraction of inspired oxygen (FiO_2_) ≥ 0.3; and (4) needing surfactant administration within 2 h after birth.

The exclusion criteria were as follows: (1) requiring intubation in the delivery room or before the application of surfactant; (2) having congenital diseases affecting respiratory function; (3) having severe congenital birth defects; and (4) having inherited metabolic disease.

In the retrospective study, porcine surfactant (Curosurf®, Chiesi, Italy) was used. Based on literature concerned on the efficacy of surfactant and our experience, 200 mg/kg of porcine surfactant was used as the first dose. A second or third dose of 100 mg/kg of porcine surfactant was used, if the infant’s condition is deteriorated and RDS was still the primary consideration.

### Data collection

The following demographic data were collected in the two groups: gestational age, birth weight, sex, mode of delivery, Apgar score at 5 min, multiple fetuses, small for gestational age (SGA), maternal complications, complete course of antenatal corticosteroids, time between birth and surfactant administration, positive end-expiratory pressure (PEEP) prior to surfactant administration, and FiO_2_ before surfactant administration.

The primary outcomes included the occurrence and severity of BPD at 36 weeks PMA or discharge, mortality before discharge, combined incidence of mortality and BPD, and mechanical ventilation (MV) rate within the first 72 h after birth. BPD was defined by the 2001 NICHD definition [10] as an oxygen supplement for at least the first 28 postnatal days, and severity was evaluated at 36 weeks PMA or at discharge, whichever occurred first. The severity of BPD was classified as mild, moderate, or severe. Indication for MV was any of the following conditions: (1) FiO_2_ ≥ 0.6 with PEEP ≥ 7cmH2O; (2) respiratory acidosis (pH ≤ 7.2); and (3) frequent apnea. The secondary outcomes included using a second or further dose of surfactants; the incidence of pulmonary hemorrhage, pneumothorax, intraventricular hemorrhage (IVH) ≥ grade III, surgical necrotizing enterocolitis (NEC), or retinopathy of prematurity (ROP) ≥ stage 3; hemodynamically significant patent ductus arteriosus (hsPDA); adverse events during surfactant administration; duration of respiratory support mode in hospitalization; and length of hospitalization.

### Statistical analysis

All statistical analyses were performed using the Statistical Package for the Social Sciences (SPSS) software, version 20.0 (SPSS, IBM, Armonk, NY, USA). Continuous variables are presented as mean with standard deviation and median with interquartile range (25th–75th percentile) and were assessed using t-tests and Mann–Whitney U-tests for normally and non-normally distributed data, respectively. Categorical variables are described using frequency and were assessed using the chi-square test or Fisher’s exact test for between-group comparison. *P*-values < 0.05 were considered statistically significant.

## Results

### Clinical characteristics

A total of 148 preterm infants of gestation age between 25^+5^ weeks and 31^+6^ weeks and of birth weights between 745 and 1490 g were diagnosed with RDS by both clinic presentations and/or chest X-ray and require surfactant replacement. Of these, 46 infants were in the LISA group; with gestational age ranged from 25^+5^ to 31^+6^ weeks and birth weight ranged from 790 to 1490 g. The remaining 102 infants were in the InSurE group; with gestational age ranged from 26^+2^ to 31^+6^ weeks and birth weight ranged from 745 to 1490 g. Other demographic and baseline clinical characteristics were similar in both groups (*P* > 0.05). The details were shown in Table 1.

**Table 1:** Participants’ demographic and baseline clinical characteristics

Variables	LISA group (n = 46)^a^	InSurE group (n = 102)^a^	*P-*value
Gestational age (weeks), mean (SD)	29.5 ± 1.4	29.1 ± 1.5	0.185
Birth weight, median (IQR), g	1245.0 (1067.5–1399.5)	1130.0 (1030.0–1402.5)	0.349
Weight at discharge, median (IQR), g	2440.0 (2267.5–2900.0)	2340.0 (2117.5–3140.0)	0.824
Male	25 (54.3)	67 (65.7)	0.188
Caesarean	37 (80.4)	76 (74.5)	0.432
Multiple fetuses	12 (26.1)	25 (24.5)	0.838
Small for gestational age	2 (4.3)	5 (4.9)	1.000
Apgar score at 5 min, median (IQR)	9 (9–10)	9 (9–10)	0.177
*Maternal complications*			
Premature rupture of membranes	14 (30.4)	37 (36.3)	0.489
Gestational diabetes	11 (23.9)	33 (32.4)	0.298
Gestational hypertension	12 (26.1)	34 (33.3)	0.378
Placental abruption	5 (10.9)	16 (15.7)	0.437
Complete antenatal corticosteroids	28 (60.9)	61 (59.8)	0.902
Time from birth to surfactant administration median (IQR), min	113.5 (93.8–241.8)	123.5 (80.0–184.5)	0.535
PEEP prior to surfactant administration median (IQR)	6.0 (6.0–7.0)	6.0 (6.0–7.0)	0.894
FiO_2_ prior to surfactant administration median (IQR)	34 (30–40)	35 (30–40)	0.553

LISA, less invasive surfactant administration; InSurE, intubate-surfactant-extubation; n, number; wk, weeks; g, grams; SD, standard deviation; IQR, interquartile range; PEEP, positive end-expiratory pressure

^a^Data are expressed as numbers (%) unless otherwise indicated

### Primary outcomes

Ten infants had BPD in the LISA group, compared with 28 infants in the InSurE group (21.8 vs. 27.4%, *P* = 0.85). There were two infants with severe BPD in LISA group, compared with four in the InSurE group (4.35 vs. 3.93% *P* = 0.903). No mortality was observed in each group. There was no significant difference in the MV rate within the first 72 h after birth, there were eight infants in the LISA group, compared with 19 in the InSurE group (8/46 vs. 19/102, *P* = 0.857). The details were shown in Table 2.

**Table 2:** Comparison of primary outcomes between the two groups

Variables	LISA group (n = 46)^a^	InSurE group (n = 102)^a^	*P*-value
MV ventilation during the first 72 h	8 (17.4)	19 (18.6)	0.857
BPD	10 (21.8)	28 (27.4)	0.462
*Severity of BPD*			
Mild	6 (13.4)	21 (20.6)	0.271
Moderate	2 (4.35)	3 (2.94)	0.438
Severe	2 (4.35)	4 (3.93)	0.903
Died during the first 28 days	0	0	NA

LISA, less invasive surfactant administration; InSurE, intubate-surfactant-extubation; BPD, Bronchopulmonary dysplasia; MV, mechanical ventilation

^a^Data are expressed as numbers (%)

### Secondary outcomes

Three infants required second or further dose of surfactant in LISA group, while 6 infants did in InSurE group. There were no significant differences between the two groups (3/46 vs. 6/102, *P* = 1.000). No infant had pneumothorax or intraventricular hemorrhage ≥ stage 3 during hospitalization in each group. There were also no significant differences in other neonatal morbidities, such as pulmonary hemorrhage, surgical NEC, hsPDA, and ROP ≥ stage3 between the groups (*P* > 0.05). There was no significant difference in the length of hospitalization between the groups (62.8 vs. 65.2 days, *P* = 0.570). The average durations of MV, nCPAP/high-flow nasal catheter, and oxygen supplementation during hospitalization were 5, 15, and 23 days in the LISA group, compared with 7, 13, and 25 days in the InSurE group, respectively (*P* > 0.05). The details were shown in Table 3.

**Table 3:** Comparison of secondary outcomes between the two groups

	LISA group (n = 46)^a^	InSurE group (n = 102)^a^	*P*-value
Second dose of surfactant or more	3 (6.5)	6 (5.9)	1.000
*Neonatal morbidities*			
Pulmonary hemorrhage	2 (4.3)	5 (4.9)	1.000
Pneumothorax	0	0	NA
Surgical NEC	1 (2.2)	0 (0)	0.311
hsPDA	15 (32.6)	32 (31.4)	0.881
IVH ≥ grade III	0	0	NA
ROP ≥ stage 3	8 (17.4)	21 (20.6)	0.650
Length of hospitalization mean (SD), days	62.8 ± 18.2	65.2 ± 22.2	0.570
*Respiratory support*			
MV days median (IQR), days	5 (1–13)	7 (1–17)	0.215
nCPAP/HFNC days median (IQR)	15 (8–22)	13 (9–30)	0.469
Oxygen days^b^ median (IQR)	23 (11–46)	25 (9–58)	0.318
*Adverse events during surfactant administration*			
Apnea	2 (4.3)	7 (6.9)	0.825
Bradycardia	3 (6.5)	9 (8.8)	0.881
Surfactant reflux	11 (23.9)	10 (10.8)	0.042

LISA, less invasive surfactant administration; InSurE, intubate-surfactant-extubation; PEEP, positive end-expiratory pressure; FIO2, fraction of inspired oxygen; NEC, necrotizing enterocolitis; hsPDA, hemodynamically significant patent ductus arteriosus; IVH, intraventricular hemorrhage; ROP, retinopathy of prematurity; nCPAP, nasal continuous positive airway pressure; MV, mechanical ventilation; HFNC: high flow nasal catheter; IQR, interquartile range; SD, standard deviation

^a^Data are expressed as numbers (%) unless otherwise indicated

^b^Oxygen days: days of hospitalization where oxygen by mask or nasal tube was required

## Discussion

In our study, it was found that LISA method was not superior to InSurE method in reducing mortality or incidence of BPD. To the best of our knowledge, it was the largest Chinese single-center observational study focusing on this issue. The main population of our retrospective study was infants at 29 weeks gestation age (mean), who were relatively older than those included in two previous multi-center studies conducted by Kribs et al. [11] and Dargaville et al. [12].

No mortality in either group was observed in our study. The result was consistent with that of Kribs et al. [11], who found that the use of LISA was not associated with mortality. However, the result was in contrast with that of a systemic review conducted by Aldana-Aguirre et al. [4], which showed that LISA could significantly reduce mortality compared with the traditional method. We speculated that the small sample size of our study and the relatively lower mortality rate of infants with gestational ages > 28 weeks would be the main reason.

We did not find significant differences in the incidence of BPD between the two groups. The result was consistent with the result of our previous study [13]. This could also be attributed to the older gestational age of our population. We speculated that LISA’s effects may depend on the population’s gestational age. Mohammadizadeh et al. [14] conducted a study including 38 neonates with an average gestational age of 30 and 31 weeks in the LISA and control groups, respectively. They found that LISA had the same effect on BPD as traditional surfactant administration. A recent study involving 40 neonates with an average gestational age of 31 weeks also reported no effects of LISA on reducing BPD, as compared with InSurE [15]. This could possibly be associated with the lower incidence of BPD in infants with gestation age > 28 weeks. A study by Ramos-Navarro et al. [16] concluded that LISA significantly decreased the incidence of BPD among neonates with gestational age 26^+0^–28^+6^ weeks, while no effect was observed among neonates with gestational ages 29^+0^–31^+6^ weeks.

It is well known that infants with severe BPD are at a higher risk of mortality after discharge [17], and there is increasing interest in identifying the relationship between LISA and BPD severity. However, there were still no consensus for the relationship from previous studies. Buyuktiryaki et al. [18] found a significant reduction in BPD severity at 36 weeks PMA in the LISA group, compared with that in the InSurE group. Recently, Dargaville et al. [12] conducted a multi-center study in infants with gestational ages between 25 and 28 weeks. They did not find a clear association between BPD severity and LISA. We speculated that many risk factors, such as gestational age, birth body weight, with prenatal/perinatal/postnatal infection and optimal managements after birth, may have different effects on the incidence of BPD. Furthermore, each factor may have various powers on the incidence of BPD among different centers.

MV is a significant risk factor for BPD in preterm infants, specifically those with very low birth weights [19, 20]. We found no significant difference in the incidence of MV within the first 72 h of birth, which was similar to the results of the study conducted by Kruczek et al. [21] In contrast, Gopel et al. [22, 23] found that LISA could reduce MV treatment within 72 h of birth for neonates with gestational ages of 26–28 weeks, as well as in his following study. A study by Kribs et al. [11] also showed that LISA could reduce the rate of tracheal intubation within the first 72 h after birth even for neonates with gestational ages < 25 weeks who were at relatively higher risk of nCPAP failure due to severe apnea or respiratory fatigue. We speculated the difference may mainly be caused by gestational age, and partly be associated with policies of MV.

Several adverse events have been observed during surfactant administration [24]. However, there were no significant differences in the incidence of adverse events between the two groups in our study. Ambulkar et al. [25] reported that approximately 5–40% of neonates undergoing LISA had adverse events, including apnea, transcutaneous oxygen desaturation, bradycardia, and choking. Surfactant reflux was also widespread during surfactant administration, especially in LISA. Furthermore, when compared with the InSurE group, the incidence of surfactant reflux was significantly higher in the LISA group. However, there was no difference in the effects of surfactant replacement, such as reduction in FiO_2_, PEEP and the number of additional surfactant doses. Thus, further studies should focus on the issue of the optimal surfactant dose for LISA.

In our retrospective study, the number of infants in the InSurE group was relatively higher than that in the LISA group. As shown in previous surveys, the doctors’ attitudes may affect the clinical application of LISA [6, 26]. Half of our cooperators were still hesitant to try this method, despite it had been using for nearly 10 years. Further researches were needed to release their concerns.

Further studies may focus on two issues. One was which population could take most advantage from the method of LISA, although it had been proven to be safe for neonates with a gestational age < 26 weeks [27]. The other was providing/incorporating the follow-up data of the survivors, who received LISA methods.

There were some limitations in our study. Firstly, our study was a single-center retrospective study, and the cohort was relatively small. Secondly, the mean gestational age in our study population was about 29 weeks, which is not the population at the highest risk for mortality and BPD. Finally, we did not perform follow-up in survivors. Our further stratified comparison studies would focus on these issues.

In our study, compared with the traditional application of surfactant administration, the LISA technique was an alternative method to delivery surfactant, but did not significantly reduce the mortality and incidence of BPD. Further researches are required to explore the potential effects of LISA on late pulmonary outcomes or neurodevelopment in survivors.

## Data Availability

The datasets used and/or analyzed during the current study are available from the corresponding author on reasonable request.

## Abbreviations

LISA: Less invasive surfactant administration
BPD: Bronchopulmonary dysplasia
MV: Mechanical ventilation
BW: Birth weight
IVH: Intraventricular hemorrhage
ROP: Retinopathy of prematurity
InSurE: Intubation-surfactant-extubation
RDS: With respiratory distress syndrome
GA: Gestational age
PEEP: Positive end-expiratory pressure
NEC: Necrotizing enterocolitis
hsPDA: Hemodynamically significant patent ductus arteriosus
